# Kabuki Syndrome With Cardiac Manifestations: A Case Report and Mini-Literature Review From the United Arab Emirates (UAE)

**DOI:** 10.7759/cureus.80981

**Published:** 2025-03-22

**Authors:** Anas Hashem, Nada K Ourfahli, Amjad M Mohamadiyeh, Amani Khalouf, Saryia Adra

**Affiliations:** 1 Department of Internal Medicine, Rochester Regional Health, Rochester, USA; 2 Department of Clinical Sciences, College of Medicine, University of Sharjah, Sharjah, ARE

**Keywords:** congenital cardiac anomalies, craniofacial dysmorphia, developmental and behavioral delay, kabuki syndrome (ks), skeletal anomalies

## Abstract

Kabuki syndrome (KS) is a rare genetic syndrome with an unknown exact etiology with suggestive autosomal dominant inheritance pattern with variable expressivity and environmental influences. KS is diagnosed based on five cardinal signs: craniofacial dysmorphia, skeletal anomalies, dermatoglyphic abnormalities, mental retardation, and postnatal growth deficiency. An eight-year-old Jordanian girl was diagnosed with KS based on characteristic clinical features at the age of four. The patient presented typical facies of KS, with elongated palpebral fissure and the eversion of the lateral part of the lower 1/3 of the eyelid; skeletal and limb abnormalities, including the early closure of the anterior fontanel; cardiogenic manifestations; global developmental delays; moderate hearing impairment; and strabismus with bilateral hyperopia. Patients with KS have various skeletal, mainly cranial, anomalies, including coronal and metopic synostosis. Cardiac malformations and aortic coarctation more commonly occur in male KS patients, supporting the X-linked hypothesis. The most common ophthalmic abnormalities in KS are strabismus and ptosis. In addition, both dental and otologic problems are common in KS. Finally, mild to moderate cognitive impairment in KS leads to significant language delays. KS is primarily diagnosed based on clinical presentation even with significant variability in the associated anomalies. In this case, we discuss the first female Kabuki syndrome (KS) patient in the United Arab Emirates (UAE) who presented with moderate to severe aortic coarctation and underwent corrective surgery with balloon dilation twice at the age of four. The patient underwent confirmatory genetic testing, identifying a heterozygous variant in the *KDM6A* gene. A multidisciplinary team including a general pediatrician, cardiologist, neurologist, orthopedics specialist, ophthalmologist, otolaryngologist, and other specialties is needed to improve the course and prognosis of KS patients and to enhance the quality of life.

## Introduction

Kabuki syndrome (KS), first described by Niikawa and Kuroki, is named after the unusual facies that resemble the makeup of actors in Kabuki, a traditional Japanese theater [1]. Initially, most of the reported cases were Japanese, and its incidence in Japan was estimated to be one in 32,000 [1]. Recently, however, studies reported a similar KS incidence rate across all ethnicities, including in Europe, the Americas, China, India, and Africa. In contrast, only three cases have been reported in the Middle East, possibly due to underdiagnosis, restricted genetic testing, and less recognizable clinical features. Additionally, the majority of reported cases were autosomal dominant heterozygous [2].

Although the exact etiology of this disorder is unknown, it is thought to be due to sporadic mutation. However, some cases of inherited transmission have been reported, with facial resemblance observed in the mothers of KS patients, which suggests an autosomal dominant inheritance pattern with variable expressivity. The absence of consistent familial occurrence and consanguinity seems to exclude recessive inheritance and suggests potential environmental influences [3]. Numerous chromosomal abnormalities, particularly in the X chromosome, have been associated with this syndrome. Additionally, nonsense and frameshift mutations in the *KMT2D* and *KDM6A* genes have been implicated in most of the affected patients, leading to abnormal chromatin regulation [4].

KS is mainly a clinical diagnosis, based on the following five cardinal manifestations: 1) a craniofacial dysmorphia (occurrence rate: 100%) characterized by elongated palpebral fissure with the eversion of the lateral part of the lower 1/3 of the eyelid, long arched eyebrows with sparse or dispersed lateral one-third, large and prominent ears, and a short nasal septum with or without a depressed nasal tip; 2) skeletal anomalies (occurrence rate: 92%), including brachydactyly of the fifth finger and a scoliosis, with or without hip luxation; 3) dermatoglyphic abnormalities (occurrence rate: 93%), including increased digital ulnar loop and hypothenar loop patterns, and the presence of fingertip pad-like swelling on all fingertips; 4) mild to moderate mental retardation (occurrence rate: 92%); and 5) postnatal growth deficiency (occurrence rate: 83%). Thus, the core of the phenotypic spectrum of KS is rather narrow and clearly defined [5]. Many other malformations have also been observed. Important among them are congenital heart defects (occurrence rate: 31%) and early breast development in infant girls (occurrence rate: 23%) [1].

Our aim is to highlight a rare genetic syndrome that is rarely reported in the Middle East, as we present the first documented case in the United Arab Emirates (UAE). Moreover, we emphasize the importance of early recognition and intervention, advocating for the active role of health authorities in ensuring access to specialized facilities and multidisciplinary care for affected patients.

## Case presentation

An eight-year-old Jordanian girl, enrolled in a special needs school in the United Arab Emirates due to mild mental retardation, was suspected to have features of KS. Her primary care physician suspected the condition at the age of four based on its characteristic clinical features. She was born to a non-consanguineous parent and has healthy siblings, with no family history of congenital anomalies, intellectual disability, or similar conditions. The mother had an uneventful pregnancy until 31 weeks of gestation when she underwent caesarean section due to a history of previous caesarean sections. Preterm labor was complicated only by maternal fever with no evidence of perinatal sepsis or intra-amniotic infection. The child, born weighing 1,800 g, was admitted to the NICU for observation and close monitoring due to prematurity and was discharged after seven days without complications. However, breastfeeding was not possible due to latching difficulty secondary to hypotonia that was noted clinically since birth, but a formal neurological assessment was not performed at that time.

**Figure 1:**
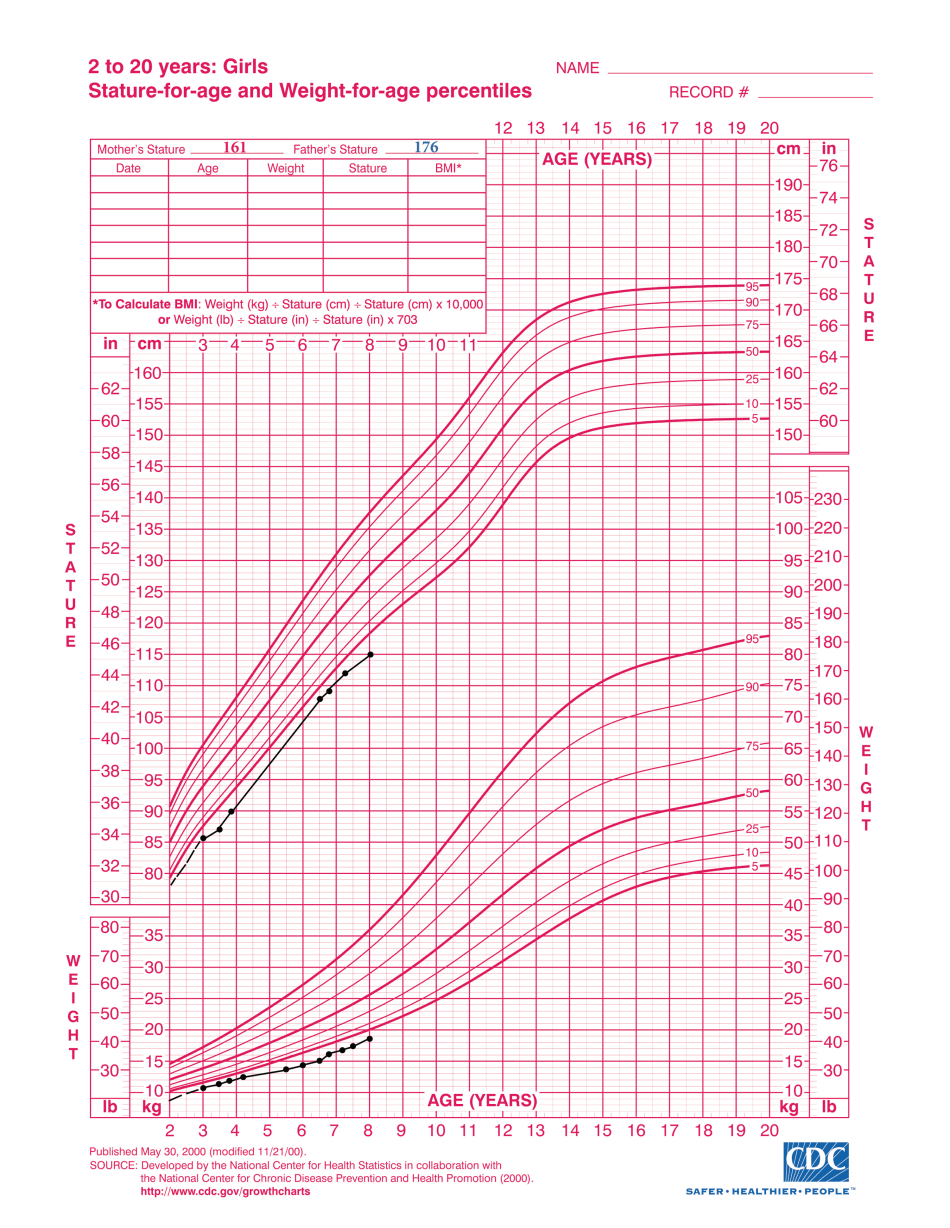
Growth chart of the patient demonstrating failure to thrive with growth parameters consistently tracking below the fifth percentile. Source: https://www.cdc.gov/growthcharts/cdc-growth-charts.htm

We assessed the patient at eight years of age. Upon physical examination, the child had failure to thrive (<5th percentile), with a weight of 19 kg, height of 115 cm (Figure 1) with postnatal dwarfism becoming apparent with age, and a small head for her age.

**Figure 2:**
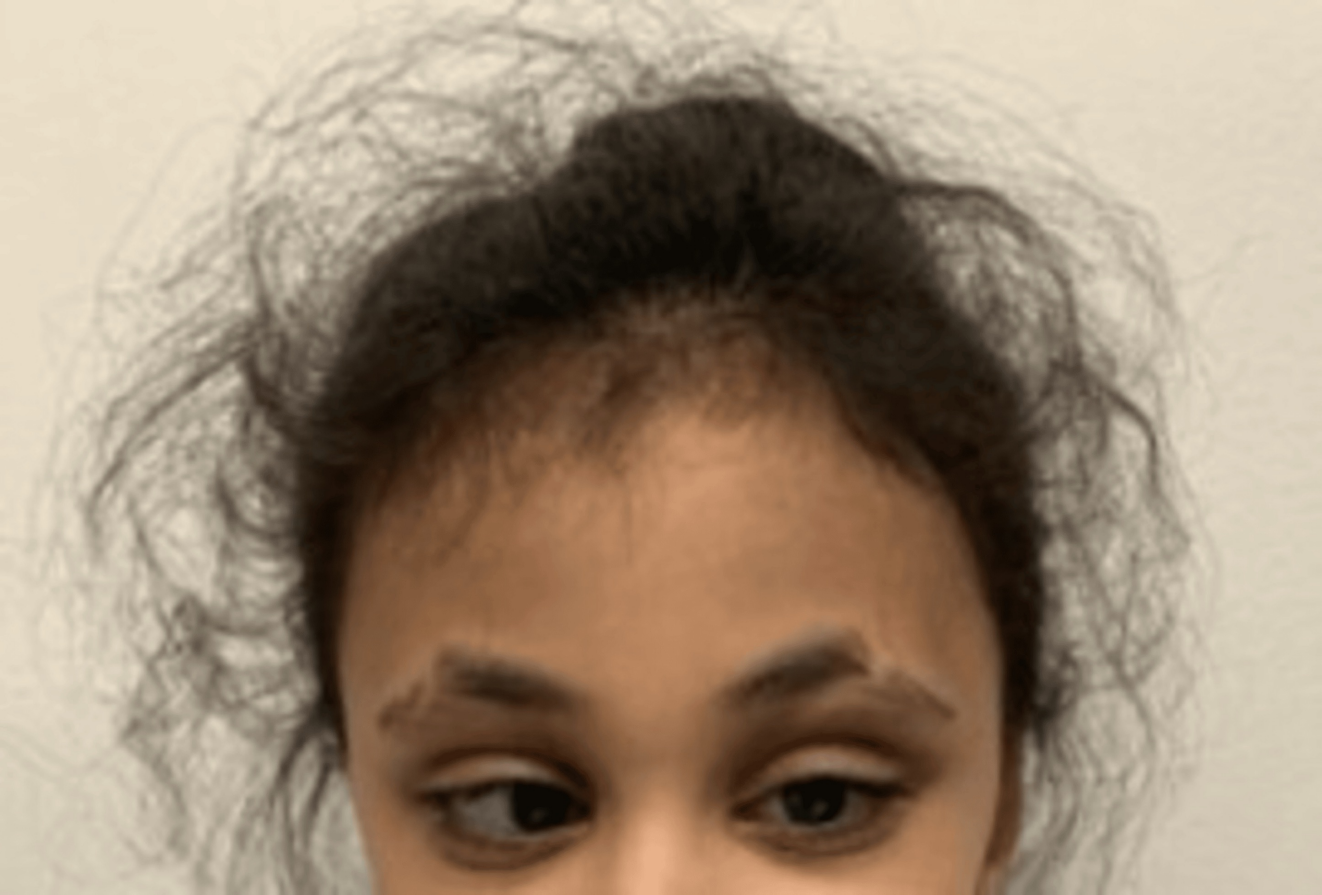
Picture of the patient exhibiting microcephaly, long arched eyebrows with sparse lateral one-third, and elongated palpebral fissure with right eye esotropia.

Besides microcephaly, the patient has the typical facies of KS, with elongated palpebral fissure and the eversion of the lateral part of the lower 1/3 of the eyelid, long arched eyebrows with sparse or dispersed lateral one-third (Figure 2), esotropia, short columella, prominent ears, and abnormal teething with black discoloration, corrected surgically at the age of four.

**Figure 3:**
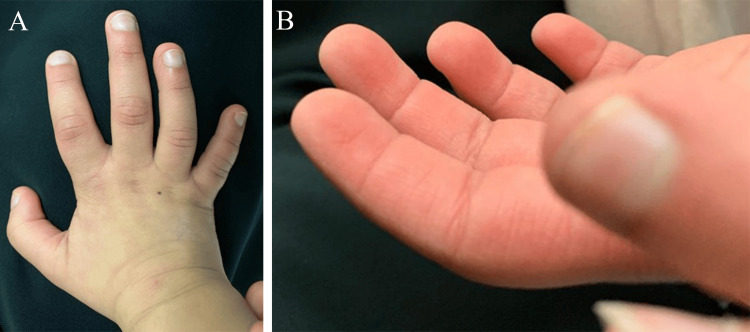
Limb anomalies in the patient. (A) Brachydactyly of all fingers except the middle finger with clinodactyly of the fourth digit. (B) Persistence of fetal fingertip pads.

The patient also had skeletal and limb abnormalities, including the early closure of the anterior fontanel, short stature, sagittal cleft vertebrae, butterfly vertebrae, narrow intervertebral disc space, and scoliosis. Additionally, she also had brachydactyly of all fingers except the middle finger and clinodactyly with radial deviation of the fourth digit (Figure 3A) with the persistence of fetal fingertip pads (Figure 3B). A bone age assessment performed at eight years of age revealed a bone age corresponding to that of a seven-year-old.

Psychomotor tests revealed a general delay in developmental milestones, with the child exhibiting a sad mood and frequently crying silently. She has been diagnosed with attention deficit hyperactivity disorder, a condition increasingly recognized as a comorbidity in KS, but shows no features of autism or self-harm [6]. Cognitive assessments indicated mild intellectual disability, with an estimated mental age of four years despite her chronological age being eight. Global developmental delays were also present as shown in Table 1.

**Table 1:** Comparison of the patient's developmental milestones with typical age ranges.

Developmental Domain	Patient's Age	Typical Age
Gross motor		
Sitting unsupported	18 months	6 months
Walking independently	2.5 years (on toes)	12 months
Riding a tricycle	7 years	3 years
Fine motor		
Pincer grasp	4 years	9-12 months
Language		
Cooing	12 months	2 months
Says "mama" and "dada"	2.5 years	9 months
Three-word sentences	5 years	3 years
Articulation difficulties	Persistent	Resolves by five years
Personal-social		
Waves bye-bye	3 years	9 months
Play preference	Prefers playing alone or with siblings	Engages with peers by five years

ENT evaluation revealed bilateral serous otitis media. A previous threshold auditory brainstem response (ABR) test, conducted six months ago, showed normal wave "V" progression at lower-intensity clicks in the right ear but poor progression in the left ear, indicating moderate hearing impairment, as determined by the auditory thresholds recorded during the test. The patient did not require a hearing aid, as the hearing loss was managed with bilateral myringotomy and grommet placement; a follow-up test showed normal hearing in both ears.

Ophthalmologic examination identified strabismus in the right eye with esotropia, as well as bilateral hyperopia (sphere +1). Visual acuity could not be assessed due to the patient's lack of cooperation during the evaluation. Additionally, medical records indicated that the patient underwent adeno-tonsillectomy eight months ago due to recurrent chronic tonsillitis.

**Figure 4:**
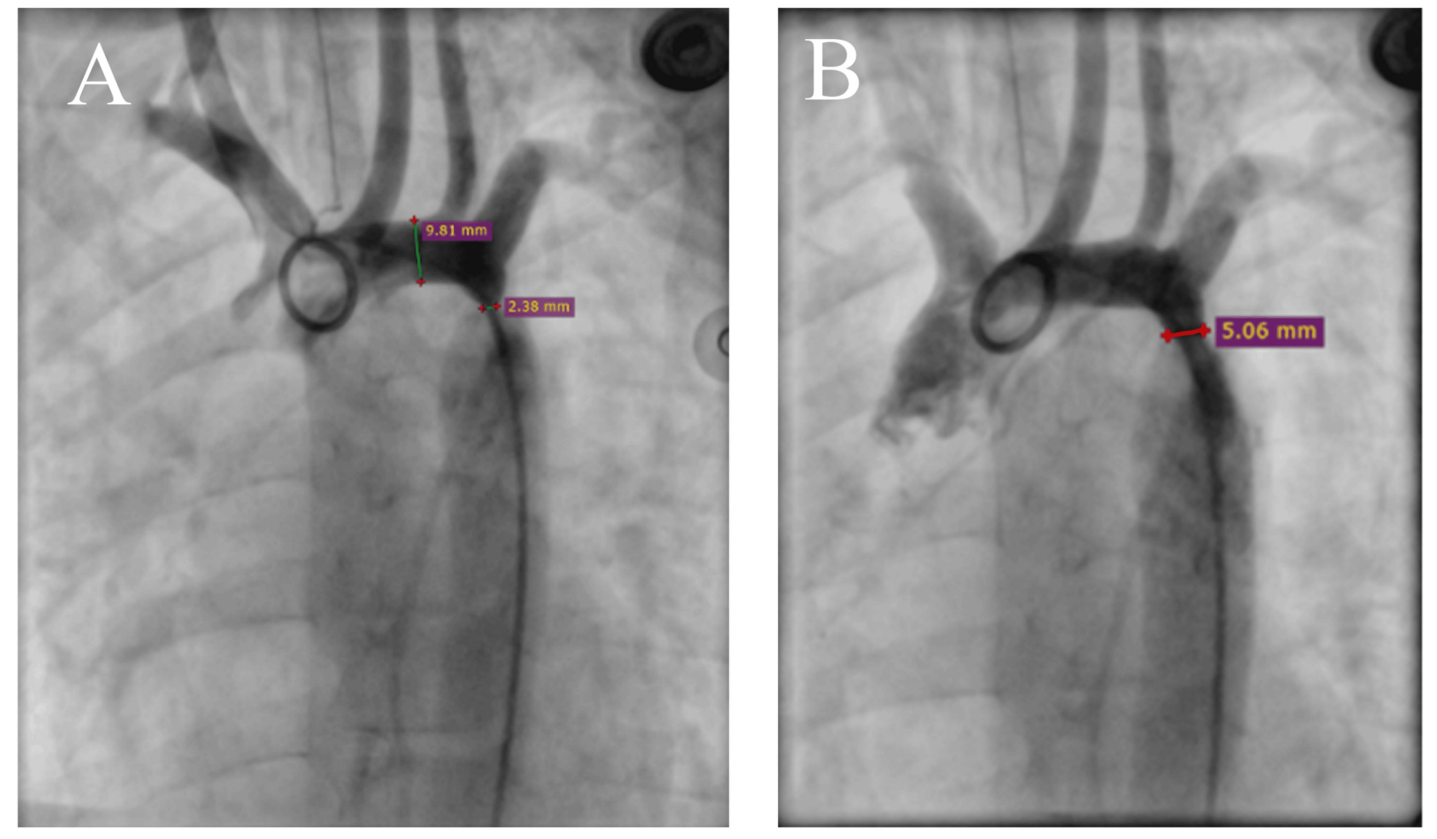
Anteroposterior aortic angiography showing moderate to severe aortic coarctation before (A) and after (B) intervention.

On auscultation, a grade 3/6 ejection systolic murmur was present in the aortic area. An echocardiogram performed at the age of four revealed mild tricuspid valve regurgitation, a parachute-like mitral valve, and mild hypertrophy of the interventricular septum, with no evidence of a ventricular septal defect or bicuspid aortic valve. The aortic measurements were as follows: ascending aorta, 12 mm; transverse arch, 7-8 mm; and a narrowed segment near the left subclavian artery, 2.6 mm, with a maximum gradient of 72 mmHg. No patent ductus arteriosus was observed. The patient was diagnosed with moderate to severe aortic coarctation and underwent corrective surgery with balloon angioplasty twice at the age of four (Figure 4). Propranolol was prescribed to reduce the risk of future complications. This cardiac presentation highlights the importance of recognizing and understanding the rare cardiac manifestation in KS.

## Discussion

Patients with KS have various skeletal and cutaneous abnormalities. Cranial anomalies, including coronal and metopic synostosis, the incomplete development of the frontal and/or maxillary sinuses, digital impressions on the skull, and the underdevelopment of the mastoid processes, are often implicated in this syndrome [3]. In our patients, early anterior fontanel closure was present, and though rarely documented, craniosynostosis is likely to be a frequent feature of this syndrome [7,8]. Ligamentous laxity and muscle hypotrophy are major pathologic factors contributing to joint dislocations in KS [9]. As in our patient, a previous case report described a KS patient with autism who also exhibited toe-walking, suggesting a possible link between KS and autistic-like behavior [10]. Although pigmented nevi have been occasionally observed in KS, our patient did not present with this feature [11].

The most common ophthalmic abnormalities in KS are strabismus (21%) and ptosis (10%) [12]. Our patient showed strabismus with esotropia in the right eye, which is consistent with multiple reported cases in the literature [12]. Hyperopia was also observed in our patient, despite its rarity in the literature. Other rare abnormalities, such as coloboma, nystagmus, microphthalmos, microcornea, corneal opacities, blue sclera, cataracts, nasolacrimal duct obstruction, caruncle lipoma, corneal pannus, retinal telangiectasia, and retinal pigmentation, have been reported in KS, but none were observed in our patient [13]. Ophthalmoscopic evaluation in our patient revealed no abnormalities in the optic nerve, retina, macula, or vascular structures, with the exception of a refractive error.

Both dental and otologic problems are common in KS, both of which were present in our case [11,14]. Our patient had conductive hearing loss, whereas individuals with KS typically have sensorineural or mixed hearing loss [15]. Vesseur et al. reported a similar case with an increased hearing threshold in the left ear; a brain MRI showed inner left ear abnormalities. That patient successfully underwent cochlear implantation [16]. As such, regular hearing monitoring is recommended for children with KS. Dental abnormalities have been reported in over 60% of patients with KS, including hypodontia (particularly of central/lateral incisors and premolars), interdental spacing, and microdontia, as well as the absence of both permanent mandibular lateral incisors, malocclusion, small dental arches, severe maxillary recession, and midfacial hypoplasia [14,17].

Cognitive impairment in KS ranges from mild to moderate intellectual disability, with our patient being on the milder aspect with significant language delays, a common feature of KS. These delays are often attributed to hearing impairments, neurological factors, or the presence of hypotonia [18]. In our case, the language delay was attributed to the patient's history of hypotonia, moderate intellectual disability, and hearing impairment prior to undergoing myringotomy and grommet placement.

Variable cardiac anomalies have also been described in KS with an incidence of 30% [19]. Based on another study on 20 KS patients, 55% (n=11) had cardiac malformations, five (25%) children had multiple malformations, and all patients had juxta-ductal aortic coarctation [20]. A more recent study of 60 KS patients found cardiac anomalies in 58% of the cases; aortic coarctation was present in 23% (n=8) of the cases and a co-occurrence of parachute mitral valve in only two patients, as in our patient. Notably, all previously reported cases of aortic coarctation have occurred predominantly in men, supporting the hypothesis of an X-linked mutation [21]; however, our case along with other documented women with KS suggests that aortic coarctation in KS is not strictly linked to male gender [22]. Given the risk of cardiac anomalies, it is crucial to monitor for related cardiac complications such as heart failure, arrythmias, failure to thrive, developmental delays, sudden cardiac events, and pulmonary hypertension, indicating the critical need for early screening and cardiac monitoring [23,24].

## Conclusions

KS was initially introduced in Japan. However, it is now known to occur worldwide among all ethnic groups and remains underrecognized by both clinicians and dentists. In this case, the patient exhibited the typical presentation of KS along with some unique features including hearing impairment, the co-occurrence of moderate to severe aortic coarctation with parachute mitral valve, hyperopia, and toe-walking. The multidisciplinary approach of genetics, cardiology, ENT, dentistry, developmental pediatrics, and other relevant specialties is needed to facilitate the improvement in the course and prognosis of these patients with the goal of promoting integral health and enhancement in quality of life. Diagnosis is primarily based on clinical presentation, with significant variability in its expression and associated anomalies among affected individuals. This diversity leads to diagnostic challenges, emphasizing the need to maintain clinical suspicion, particularly in atypical cases. The outcome and future prognosis of these patients can be better understood by identifying and tracking more cases, which can be achieved by national and international registries establishing standardized protocols for early detection through genetic testing and diagnostic criteria, along with guided treatment and global research engagement. A multidisciplinary approach is essential for optimizing patient management, improving disease outcomes, and ultimately enhancing the quality of life for individuals with KS.
